# Genome Sequence of *Torulaspora delbrueckii* NRRL Y-50541, Isolated from Mezcal Fermentation

**DOI:** 10.1128/genomeA.00438-15

**Published:** 2015-07-23

**Authors:** Jorge Gomez-Angulo, Leticia Vega-Alvarado, Zazil Escalante-García, Ricardo Grande, Anne Gschaedler-Mathis, Lorena Amaya-Delgado, Javier Arrizon, Alejandro Sanchez-Flores

**Affiliations:** a Centro de Investigación y Asistencia en Tecnología y Diseño del Estado de Jalisco, AC, Unidad de Biotecnología Industrial, Jalisco, Mexico; b Centro de Ciencias Aplicadas y Desarrollo Tecnológico, UNAM, Distrito Federal, Mexico; c Unidad de Secuenciación Masiva y Bioinformática, Instituto de Biotecnología de la UNAM, Morelos, Mexico

## Abstract

*Torulaspora delbrueckii* presents metabolic features interesting for biotechnological applications (in the dairy and wine industries). Recently, the *T. delbrueckii* CBS 1146 genome, which has been maintained under laboratory conditions since 1970, was published. Thus, a genome of a new mezcal yeast was sequenced and characterized and showed genetic differences and a higher genome assembly quality, offering a better reference genome.

## GENOME ANNOUNCEMENT

*Torulaspora delbrueckii,* an ascomycetous yeast, has high osmotolerance and high freeze tolerance (1–4). These properties make this yeast an interesting organism, with potential biotechnological applications in bakery, wine, and dairy industrial processes. In the last few years, studies about enzyme production in winemaking have been carried out, but only a few genes have been characterized (5). Recently, it has been reported that *T. delbrueckii* yeast strains isolated from the mezcal-fermenting process produced β-fructofuranosidase enzymes with fructosyltransferase activity (6). Therefore, the genome sequencing of *T. delbrueckii* can lead to the discovery of new genes with biotechnological application. Lately, the genome of *T. delbrueckii* CBS 1146 was obtained (using 454 sequencing), with the main purpose of studying the sex chromosome evolution in the *Saccharomycetaceae* family (7). However, the characterized strain has been maintained under laboratory conditions since 1970. Thus, in order to find the differences between the published reference and our mezcal isolate, we sequenced, assembled, and characterized it in order to find genes and variations associated with the fermentation process.

Genomic DNA from *T. delbrueckii* NRRL Y-50540 was isolated and prepared as Illumina sequencing libraries to generate a total of 20,514,013 paired-end reads (estimated coverage, ~328×) with a length of 72 bases, using the Illumina GAIIx platform. The assembly was performed with Velvet version 1.2.10 using a *k*-mer size of 35 (8). An assembly of 11,236,894 bp in 374 contigs with length ≥1,000 bp was obtained, with *N*_50_ and *N*_90_ values of 82,617 and 23,849 bp, respectively. The average contig length was 30,012 bp, giving a considerable space to search for genes. Finally, we ordered and scaffolded the assembly using ABACAS (9) against the available *T. delbrueckii* reference genome (7) of a different strain to leave the whole assembly in 8 scaffolds corresponding to 8 chromosomes. The average G+C content was 42%, which is consistent with the reported genome. Gene prediction was performed using AUGUSTUS version 2.7, and using several different yeast species profiles, we predicted 4,714 protein-coding genes by intersecting all predictions (10). Using CEGMA version 2.5, we obtained a 97% genome completeness (11).

In contrast to the genome published by Gordon et al. (7), we found a slightly better value for completeness and fewer open reading frames (ORFs) (4,714 versus 4,972, respectively) in our assembly. Both assemblies presented gaps, but we were able to remove some of them, which led to a more complete genome. Although these differences are not of concern, they are expected, since each genome was assembled using different sequencing technologies and assembly strategies. Currently, we are working on a hybrid assembly strategy using the information from both strains in order to obtain a better assembly, gene prediction, and annotation.

We believe that the *T. delbrueckii* genome sequence presented here can be used as a better reference to perform further analyses, such as differential gene expression of enzymes related to the synthesis and degradation of biotechnological molecules of interest, for example, under different fermentation conditions analyzed using RNA sequencing (RNA-seq) data.

### Nucleotide sequence accession numbers.

This whole-genome shotgun project has been deposited at the NCBI GenBank database under the accession numbers CP011778 to CP011785.
